# Rapid Detection of *Actinobacillus pleuropneumoniae* From Clinical Samples Using Recombinase Polymerase Amplification

**DOI:** 10.3389/fvets.2022.805382

**Published:** 2022-03-25

**Authors:** Oliver W. Stringer, Yanwen Li, Janine T. Bossé, Matthew S. Forrest, Juan Hernandez-Garcia, Alexander W. Tucker, Tiago Nunes, Francisco Costa, Preben Mortensen, Eduardo Velazquez, Paul Penny, Jesus Rodriguez-Manzano, Pantelis Georgiou, Paul R. Langford

**Affiliations:** ^1^Section of Paediatric Infectious Disease, Department of Infectious Disease, Imperial College London, London, United Kingdom; ^2^Wolfson College, University of Cambridge, Cambridge, United Kingdom; ^3^Department of Veterinary Medicine, University of Cambridge, Cambridge, United Kingdom; ^4^Ceva Animal Health Ltd., Saúde Animal, Algés, Portugal; ^5^Ceva Animal Health Ltd., Libourne, France; ^6^Ceva Animal Health Ltd., Amersham, United Kingdom; ^7^Section of Adult Infectious Disease, Department of Infectious Disease, Imperial College London, London, United Kingdom; ^8^Department of Electrical and Electronic Engineering, Imperial College London, London, United Kingdom

**Keywords:** *A. pleuropneumoniae*, RPA (recombinase polymerase amplification), *apxIVA*, point-of-care (POC), FTA^®^ card

## Abstract

*Actinobacillus pleuropneumoniae* (APP) is the causative agent of porcine pleuropneumonia, resulting in high economic impact worldwide. There are currently 19 known serovars of APP, with different ones being predominant in specific geographic regions. Outbreaks of pleuropneumonia, characterized by sudden respiratory difficulties and high mortality, can occur when infected pigs are brought into naïve herds, or by those carrying different serovars. Good biosecurity measures include regular diagnostic testing for surveillance purposes. Current gold standard diagnostic techniques lack sensitivity (bacterial culture), require expensive thermocycling machinery (PCR) and are time consuming (culture and PCR). Here we describe the development of an isothermal point-of-care diagnostic test - utilizing recombinase polymerase amplification (RPA) for the detection of APP, targeting the species-specific *apxIVA* gene. Our APP-RPA diagnostic test achieved a sensitivity of 10 copies/μL using a strain of APP serovar 8, which is the most prevalent serovar in the UK. Additionally, our APP-RPA assay achieved a clinical sensitivity and specificity of 84.3 and 100%, respectively, across 61 extracted clinical samples obtained from farms located in England and Portugal. Using a small subset (*n* = 14) of the lung tissue samples, we achieved a clinical sensitivity and specificity of 76.9 and 100%, respectively) using lung imprints made on FTA cards tested directly in the APP-RPA reaction. Our results demonstrate that our APP-RPA assay enables a suitable rapid and sensitive screening tool for this important veterinary pathogen.

## Introduction

*Actinobacillus pleuropneumoniae* (APP) is a highly contagious respiratory pathogen and the causative agent of porcine pleuropneumonia. It is one of the most frequently identified bacterial agents causing porcine respiratory infections (1), and is responsible for high economic losses to the swine industry worldwide (2). The clinical symptoms of APP infection are often indistinguishable from other bacterial and viral respiratory infections, with clinical signs including a decrease in growth rate, breathing difficulties, fever, and high mortality (1, 3).

Economic losses incurred by APP infection are largely due to increased mortality rates, animals requiring a longer time to reach their finishing weight, and costs incurred by treatment and metaphylaxis (1, 2). APP responds well to antibiotics, however, the spread of antimicrobial resistance genes has been extensiively reported and may limit the efficacy of treatment (4–6). Therefore, there is an ongoing need for continued disease surveillance and rapid diagnosis to reduce antibiotic usage and losses incurred to the industry.

There are geographical differences in the seroprevalence of the currently identified 19 serovars of APP (7, 8), with serovar 8 predominant in the UK (9). Production of certain key virulence factors vary between different serovars, which can influence the severity of disease and affect vaccine efficacy. Typically, isolates of a given serovar produce one or two of three different Apx toxins, with a fourth (ApxIV) produced by all APP isolates. Surveillance for the presence of the pathogen, as well as detection of predominant serovars, is important for biosecurity measures as well as determination of appropriate disease mitigation strategies. Historically, serotyping was performed using serological methods, with detection of specific capsule antigens determining the serovar. However, similarities between lipopolysaccharide O-antigens amongst subsets of serovars (e.g., 1/9/11, 3/6/8/15, and 4/7) can result in cross-reactions (10–12), thus a shift toward more robust molecular serotyping has occurred (13).

The current gold standard molecular diagnostic technique is the polymerase chain reaction (PCR), typically targeting the species-specific ApxIV toxin encoding gene, *apxIVA*. However, PCR is costly and time-consuming, requiring 2–3 hours to complete with highly trained personnel and complex equipment. Numerous PCR and qPCR assays with high sensitivity and specificity have been described for the detection of APP (14–16). Isothermal amplification techniques rely on enzymes to denature DNA, facilitating primer annealing and subsequent amplification of target sequences at a single temperature. One such isothermal technique is recombinase-polymerase-amplification (RPA), which utilizes T4 bacteriophage enzymes (UvsX, UvsY, and Gp32) to anneal primers to their complementary sequence within DNA (17). An advantage of RPA over other isothermal techniques is the requirement of only two primers, similar to PCR. Typically, RPA primers are longer in length than PCR primers, as increased primer length (>28 bp) has been found to increase the rate of amplification as UvsX exhibits a higher rate of ATP hydrolysis when forming filaments with longer oligonucleotides (17, 18). RPA amplification can be monitored in real-time with the addition of an exonuclease probe, with several sensitive and specific RPA assays previously described for porcine respiratory pathogens (19–22). Fluorometers are commercially available in a small form, portable device, for a fraction of the cost of a complex thermocycler. Furthermore, RPA reagents are available in a lyophilized format, facilitating stable transportation, making RPA the most versatile point-of-care molecular diagnostic technique currently available.

A significant difficulty in the implementation of a simple and rapid point-of-care test is the process of sample preparation. Labor intensive spin column methods (for binding, purification, and elution of nucleic acids) require high-speed centrifugation, which is unsuitable for use in a field setting. We have previously described an alternative sampling method, directly imprinting infected tissue on FTA cards, which chemically lyse and entrap nucleic acids. We have shown the effectiveness of this method, when combined with a simple water wash and our multiplex PCR, for the detection and serotyping of APP (23). Further adaptation of this sample preparation for combination with RPA could prove a powerful tool to screen for APP infection on-site, i.e., directly at the farm or abattoir.

In this study, we describe an RPA assay, initially targeting the *apxIVA* gene for detection of all APP (APP-RPA), regardless of serovar, with validation of the assay using 61 extracted clinical samples, 17 lung homogenates and a small subset (*n* = 14) of lung imprints made on FTA cards.

## Materials and Methods

### RPA Primer Design

The APP species-specific *apxIVA* gene was used to design APP-RPA primers and probe. The *apxIVA* gene from serovar 8 strain MIDG2331 (GenBank: LN908249.1, nt: 1,131,104-1,136,935) was subjected to a BLAST 2.0 search against the available genome assemblies of APP (taxid ID: 715) available in the GenBank database (https://www.ncbi.nlm.nih.gov/genbank/). The resulting consensus sequence when mapped to the MIDG2331 genome matched an alternative gene annotated *hemolysin A* (nt: 1,124,268-1,128,977), but has subsequently been reported as a partial duplication of the *apxIVA* gene, denoted *apxIVS* (24). For simplicity, we will refer to the *apxIVS* sequence of MIDG2331 as *apxIVA*, as the *apxIVA* consensus sequence from multiple APP isolates showed a higher sequence similarity to *apxIVS* than *apxIVA* in MIDG2331. Originally we designed an RPA assay against the same target region of *apxIVA* that is amplified in the qPCR assay described by Tobias et al. (16). However, this was subsequently found to result in several alternative priming sites due to being located within a repetitive region of the *apxIVA* sequence. Therefore, a 49 bp probe was designed within a conserved region located at the 3' end of the *apxIVA* sequence (nt: 1, 124, 315-1, 124, 454), in addition to 10 forward and 10 reverse 35 bp primers, designed in the flanking region of the probe in accordance with the manufacturer's instructions (TwistDx, Maidenhead, UK). In silico analysis of primer dimerization was assessed using the Thermo Fisher multiple primer analyzer tool (Thermo Fisher UK, Loughborough, UK).

To achieve optimal performance, primer combinations were experimentally screened in duplicate against 200 copies/μL of the serovar 8 reference strain 405 (25). The primer pairs displaying the highest sensitivity are shown in Table 1, covering a 140 bp region of the *apxIVA* gene (Supplementary Figure 1).

**Table 1:** RPA primers and probes used in this study.

**Name**	**Sequence 5^**′**^-3^**′**^**	**Amplicon size (bp)**
APP-RPA_F	GCGACACAAGAGATATCTCTCCTCCGTGCTTCTGA	140 bp
APP_RPA_R	GTATTCACACCAAGATCATAAAATAGAAAATATTC	
APP-RPA_Prb	AAACGTTGGTGAGCACTCAGGTGGAGAAGA[T(FAM)[dspacer]G[T(BHq-1)]TGAGTCGATGGCCGG	N/A

*Optimized primers for the detection of the APP specific apxIVA gene*.

### RPA Reaction Conditions

RPA reactions were performed using the TwistAmp Liquid Basic kit (TwistDx, Cat #TALQBAS01), according to the manufacturer's recommendations, with final concentrations of 0.6 μM of each primer (Thermo Fisher UK, Cat #10336022), 0.12 μM probe (LGC Biosearch Technologies, Risskov, Denmark, Cat #RPA-BF-2), 2 U/μL *Escherichia coli* Exonuclease III (NEB, Hitchin, UK, Cat #M0206L), 14 mM MgAc, and 1 μL of DNA template, in a total of 5 μL/reaction for limit of detection and specificity experiments. For extracted clinical samples and FTA card amplifications, 25 μL reactions were used, since their use yielded more consistent results over 5 μL reactions. Amplification was visualized with a Bio-Rad CFX Connect Real-Time PCR detection system (Bio-Rad, Cressier, Switzerland, Cat #1855201), running for 20 min at 37°C, with a pause at 4 min where the reactions were manually agitated (by inversion 10-times), a step which is favorable for reaction kinetics (26). Fluorescence data acquisition was taken once every 30 s (i.e., once per cycle) for a total of 40 cycles (20 min).

### RPA FTA Card Reaction Conditions

RPA reagent concentrations remained as above, however reaction volumes were increased to 25 μL to account for the FTA card disc. Samples on FTA classic cards (Whatman plc, Little Chalfont, Buckinghamshire, UK, Cat #WB120205) were prepared by pressing the card surface against lung tissue as previously described (23). FTA cards were allowed to dry fully prior to being processed, 3 mm discs were removed from the inoculated card with a sterile biopsy punch (Integra Miltex Cat #12-460-406, Fisher Scientific, Loughborough, UK), which was rinsed in ethanol and deionized water between uses to prevent cross-contamination. The 3 mm discs were washed twice for 5 min in deionized water and added directly to the 25 μL RPA reaction for amplification.

### RPA Lung Homogenate Reaction Conditions

RPA reagent concentrations remained as above. 50 mg of lung tissue was excised with a sterile scalpel, placed in a 2 mL lysing matrix A microcentrifuge tube (MP Biomedicals, Cat #116910050-CF) along with 400 μL of distilled water. The sample was then homogenized using a FastPrep-25 5G (MP Biomedicals, Cat #116005500) set at 6.0 m/s for 60 s. The microcentrifuge tube was left to settle at room temperature for 5 min prior to the supernatant being removed and used in subsequent 25 μL APP-RPA reactions.

### Extraction and Quantification of Genomic DNA

APP reference strains of serovars 1–19 (i.e., strains 4074, S1536, S1421, M62, L20, Femø, WF83, 405, CVJ13261, D13039, 56153, 1096, N273, 3906, HS143, A-85/14, 16287-1, 7311555, and 7213384-1, respectively) and two APP clinical isolates, with transposons interrupting or flanking their *apxIVA* genes, one designated as capsule type K3:O7 and one serovar 15 (MIDG2206 and MIDG 3936, respectively) were used in this study. APP isolates were grown overnight on Bacto Brain Heart Infusion broth (BD, Berkshire, England, UK, Cat #2237500) containing 1.5% agar (Sigma-Aldrich, Gillingham, Dorset, UK, Cat #W201201) and supplemented with 0.01% nicotinamide adenine dinucleotide (MP Biomedicals, Eschwege, Germany, Cat #100319). Genomic DNA (gDNA) was extracted from bacterial cells harvested from the plate cultures. Briefly, half a 10 μL loop of bacterial colonies were resuspended in 200 μL of PBS and DNA extraction was performed using a FastDNA spin kit (MP Biomedicals, Cat #116540600-CF) according to the manufacturer's instructions. All extracted gDNA was quantified using a Nanodrop-1000 spectrophotometer (Thermo Fisher UK, discontinued) and run on a 1.5% w/v agarose gel to ensure genomic integrity.

### DNA Extraction From Clinical Tissues

A total of 61 clinical lung samples, were obtained from farms in England (*n* = 36) and Portugal (*n* = 25) with a history of APP infection. Lung samples were obtained during necropsies from pigs displaying clinical signs of infection, suspected to be APP. For the extraction of lung tissue, ~50 mg of defrosted tissue were collected with a sterile disposable scalpel and homogenized in a lysing matrix A 2-mL microcentrifuge tube with 360 μL ATL buffer (Qiagen Ltd., Manchester, UK, Cat #19076) using a FastPrep-25 5G set at 6.0 m/s for 60 s. Subsequent DNA extraction was performed with a QIAmp DNA Mini Kit (Qiagen Ltd., Cat #51304) and eluted using 200 μL buffer AE (Qiagen Ltd., Cat #19077).

Five oral fluid samples were obtained from farms in England. Each oral fluid sample was extracted from a cotton rope, with each rope used to sample 25 animals at a time. Each rope was individually extracted using a MagMAX Express-96 Particle Processor (Thermo Fisher UK, Cat #4400074), as previously described (27).

### Analytical Sensitivity and Specificity

The APP-RPA assay performance was assessed using gDNA from APP serovar 8 strain 405 diluted to give between 2 x 10^5^ to 0.5 copies/μL in 10-fold serial dilutions made in TE buffer. Further dilutions of gDNA in TE buffer were made in smaller intervals between detection limits. Limit of detection experiments using serovar 8 strain 405 were run using two separate dilutions of template, with 12 replicates of each condition tested alongside no template controls. The APP-RPA assay sensitivity was additionally assessed using gDNA from the reference strains of all currently known (*n* = 19) APP serovars, each at 200 copies/μL.

The APP-RPA assay specificity was assessed by amplifying duplicate APP-RPA reactions with 10 ng of gDNA obtained from eight other bacterial species (*n* = 27), including bacteria commonly found to occupy the same niche as APP, as well as other members of the *Pasteurellaceae* family, as previously described (7). All species tested for specificity are shown in Supplementary Table 1.

### APP Quantification of Clinical Samples

Designation of the 61 extracted clinical samples as APP-positive or APP-negative was determined by qPCR targeting the APP species-specific *apxIVA* gene designed and validated by Tobias et al. (16). qPCR reactions containing 0.5 μM of each primer (Thermo Fisher UK, Cat #10336022), 0.3 μM Taqman probe (Sigma-Aldrich, UK, Cat #VC0023N) and 1x GoTaq probe qPCR master mix (Promega, Southampton, UK, Cat #A6102) for a total reaction volume of 5 μL. Reactions were run in a Bio-Rad CFX Connect Real-Time PCR thermocycler (Bio-Rad, Cat #1855201) with the following cycling conditions: 2 minutes at 95°C, followed by 40 cycles of 15 s at 95°C and 1 min at 60°C, with data acquisition taken at each cycle.

A qPCR standard curve was obtained from Ct values obtained from 10-fold serial dilutions of serovar 8, strain 405, amplified by qPCR in triplicate across three separate dilutions. The standard curve (semi-log linear regression line) was calculated using GraphPad Prism version 9.2.0 for Mac (GraphPad Software, San Diego, California USA). Relative copy numbers of APP present in clinical samples were estimated by extrapolation from the standard curve using two Ct values obtained from each clinical sample, within a 95% confidence interval.

The clinical performance of the APP-RPA assay was assessed using the extracted clinical samples, each tested in duplicate. Samples were scored as APP-RPA positive if both replicates overcame the threshold fluorescence level, whilst samples were scored as APP-RPA-negative if one or both replicates failed to reach the threshold fluorescence level.

## Results

### APP-RPA Sensitivity

Twenty APP-RPA primer combinations were screened using gDNA from the serovar 8 reference strain 405 at 200 copies per μL reaction (data not shown), and the best performing primer pair was taken forward to assess the sensitivity of the APP-RPA assay. The chosen primer pair, APP-RPA_F and APP-RPA_R, amplified a 140 bp sequence that was well conserved, as assessed by BLAST searches against all available APP genomes, with 100% pairwise identity and 97.9% identical sites across 100% sequence coverage. The partial duplication seen in some APP isolates (24) results in an APP-RPA alternative priming site in these isolates (Supplementary Figure 1).

**Figure 1:**
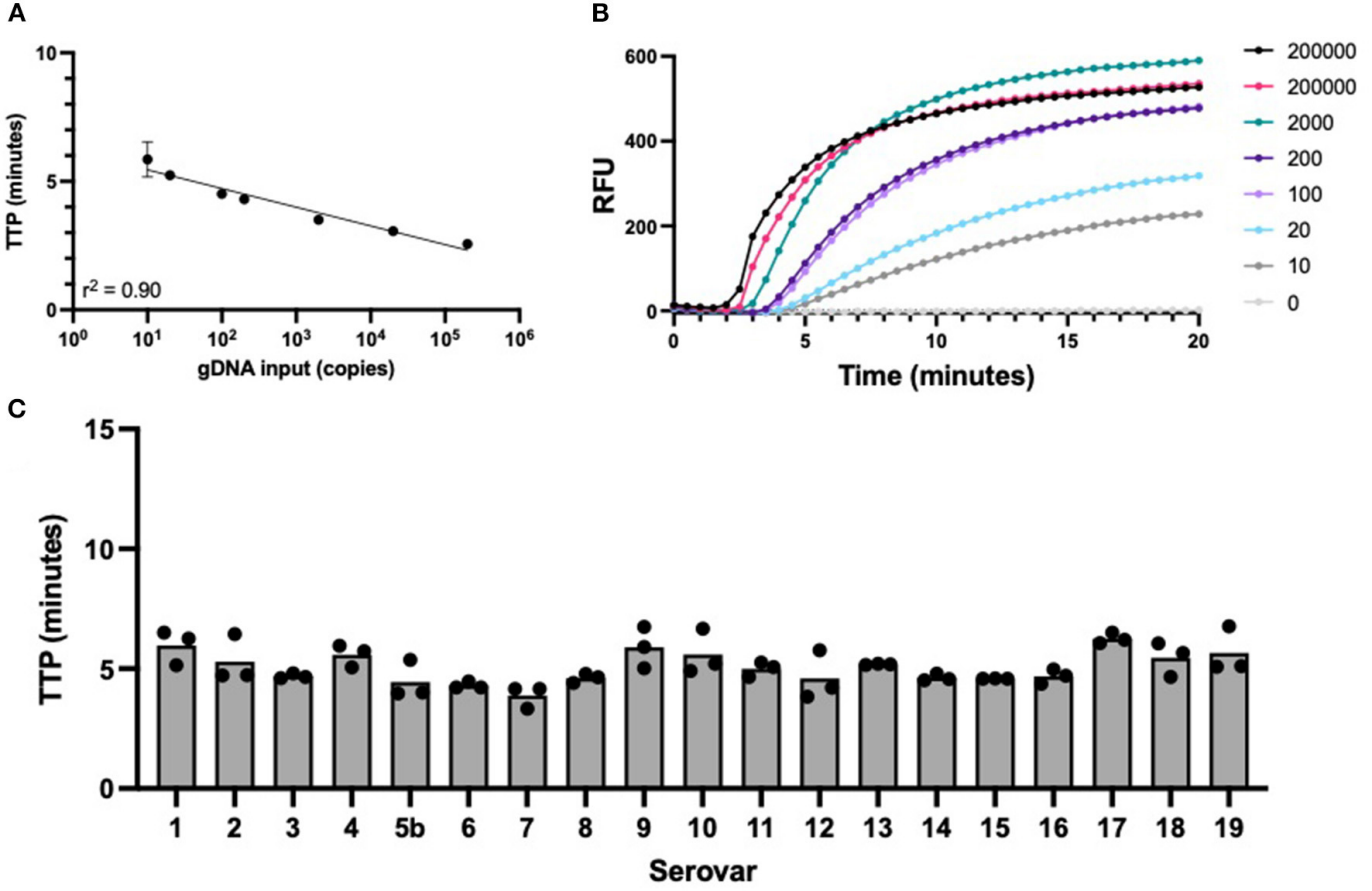
APP-RPA performance. **(A)** Regression line of APP-RPA limit of detection across 12 replicates using two different template dilutions of serovar 8 reference strain 405. Equating gDNA input to time to positive (TTP). **(B)** Fluorescence data of the amplification of APP-RPA, calculated from the average relative fluorescence units (RFU) obtained from 12 replicates of APP serovar 8. **(C)** TTP of all known serovars of APP reference strains amplified in APP-RPA at 200 copies/μL.

The APP-RPA assay sensitivity was assessed using gDNA from the serovar 8 reference strain 405, as this serovar is the most common cause of clinical pleuropneumonia in England and Wales (9). The APP-RPA assay achieved a sensitivity of 10 gDNA copies per μL reaction (Figures 1A,B), with all positive amplification occurring in under 10 min (Figure 1C).

Two APP clinical isolates which had previously undergone whole genome sequencing (WGS) (unpublished data) showing they contain transposases, within or adjacent to their *apxIVA* genes were also used (Supplementary Figure 1). MIDG2206 originated from Denmark and designated as having a K2:O7 capsule type, contains a hypothetical protein (WP_017357847) between *apxIVA* and *lacZ*, with the sequence immediately 3′ to the hypothetical protein being identical to the 3′ end of *apxIVA* resulting to an alternative priming site for the APP-RPA assay. MIDG3936, a serovar 15 isolate, contains a fragmented *apxIVA* with three ORFs, with the longest being 3867 bp. Additionally, this isolate contains a partial duplication of *apxIVA*, designated as *apxIVS'*, with a transposon flanking the duplication (WP_005599960). These isolates were verified to be detected with APP-RPA. Both MIDG2206 and MIDG3936 displayed a similar onset time at 200 copies/μL as their reference strain serovars, serovar 2 and serovar 15, respectively (data not shown). Therefore, isolates with the WP_017357847 hypothetical protein between the *apxIVA* and *lacZ* genes, or a fragmentation pattern of *apxIVA* similar to that seen in MIDG3936, are unlikely to interfere with their positive APP identification using RPA-APP.

### APP-RPA Specificity

The sequence of the APP-RPA amplicon (140 bp) was used in a BLASTn interrogation of the NCBI nucleotide database (excluding APP), with no significant similarity found for any species, indicating that only APP should cause amplification to occur. Additionally, we experimentally tested gDNA from 27 isolates, including 8 different species found in the same host niche as APP, as well as related members of the *Pasteurellaceae* family. None of these bacterial species caused detectable amplification with the APP-RPA primer set (Supplementary Table 1). Taken together, these results suggest that our APP-RPA assay is highly specific, detecting only the APP species-specific gene, *apxIVA*.

### Clinical Sensitivity

**Figure 2:**
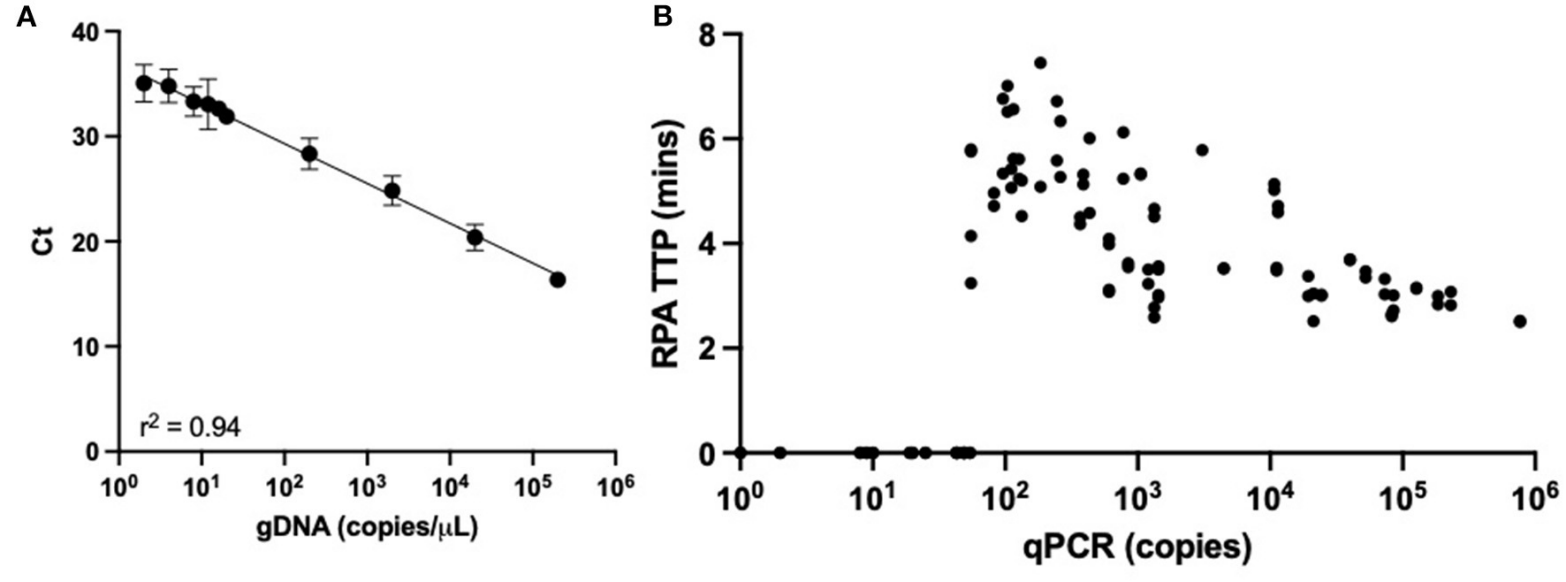
APP-qPCR performance. **(A)** Regression line from limit of detection of APP-qPCR performed with gDNA from serovar 8 reference strain 405. **(B)** Relative quantification of APP copy burden for 61 clinical samples with respective APP-RPA time to positive (TTP).

The 61 clinical samples with suspected APP infections were obtained from farms throughout Europe (UK: *n* = 36, Portugal: *n* = 25). Extracted gDNA from these clinical samples were characterized in *apxIVA*-qPCR to determine APP infection status, along with the relative bacterial load. For this, qPCR standards were performed using gDNA from serovar 8 strain 405, with the assay proving highly sensitive, being able to consistently detect 10 gDNA copies per μL reaction (Figure 2A). Of the 61 clinical samples, 51 were positive for APP by qPCR and 10 were negative, with samples giving a relative quantification value below the limit of detection considered negative. The relative quantification of clinical samples showed a range of copy numbers were present, from 11 to 10^6^ copies (Figure 2B).

In order to assess our APP-RPA performance, the APP-qPCR characterization was compared to the results obtained with the same clinical samples tested in our APP-RPA assay. Only samples displaying positive amplification in both replicates were considered positive. Samples showing a low burden (<200 copies) by qPCR performed poorly in APP-RPA, with only 10 of the 18 samples amplifying, giving a sensitivity of 55.6%. Of these low burden APP-positive samples, APP-RPA detected all four samples from oral fluid, and six out of 14 extracted from lung tissue. All the low burden samples missed by APP-RPA were quantified by qPCR as having a bacterial load lower than 55 copies (equating to less than 2.2 copies per μL in the RPA reaction). For samples characterized as having a medium bacterial burden (201–2,000 copies) by qPCR, APP-RPA detected all 15 of the samples, including one oral fluid sample, equating to a sensitivity of 100%. The one oral fluid sample in the medium bacterial load group contained 260 copies, determined by qPCR. Of the high burden samples (>2,001 copies), our APP-RPA detected all 18 of the samples, therefore, the high burden samples gave a sensitivity of 100%. All samples in this burden category were derived from lung tissue. Overall (i.e., across all burden levels), our APP-RPA assay achieved a sensitivity of 84.3%, with 100% specificity (Table 2).

**Table 2:** Performance of APP-RPA in 61 clinical samples.

**Bacterial load (Relative quantification)**	**qPCR**			**APP-RPA**	
	**Upper qPCR limit (Ct)**	**Lower qPCR limit (Ct)**	**Total number (*n*)**	**Sensitivity (%)**	**Specificity (%)**
High	N/A	27.28	18	100	N/A
>2,001 copies					
Medium	27.27	31.01	15	100	N/A
201–2,000 copies					
Low	31.00	35.64	18	55.6	N/A
<200 copies					
Negative	35.63	N/A	10	N/A	100
Total	N/A	35.64	61	84.3	100

*Sixty one clinical samples (56 lung samples and 5 oral fluid samples) were obtained from various farms, 36 in England and 25 in Portugal. Clinical samples were characterized in qPCR and relative quantification was performed based on Ct values. Subsequently, samples were run in APP-RPA to assess the clinical performance of the assay*.

### FTA Card Clinical Comparison

To evaluate the use of lung smears made on FTA cards as a sample isolation method amenable to rapid a diagnostic test for APP, a subset of frozen lung samples (*n* = 13) were inoculated onto FTA cards, which were then dried and washed using our previously described water-wash protocol validated for use in PCR (23). In the clinical evaluation above, one of the lungs were scored as having been negative for APP, three of the lungs were determined to contain low, four medium and five high bacterial loads by qPCR. The overall sensitivity of the FTA-APP-RPA was 75%. However, of the three samples with low bacterial loads (i.e., 96, 127, and 185 copies, as quantified by qPCR) two amplified with the FTA-APP-RPA, with the lowest bacterial load (96 copies) being the sample that was missed by FTA-APP-RPA. Conversely, FTA-APP-RPA failed to detect a sample displaying medium and a high bacterial burden, from lung tissue that was quantified as having 572 and 82,770 copies by qPCR.

### Homogenized Lung Clinical Comparison

In order to achieve more rapid detection of APP, the use of crude homogenized tissue was assessed. Lung samples (*n* = 16) that had previously been subjected to genomic extraction and relative quantification of APP by qPCR also underwent homogenization. Lung sections were dissected, suspended in 400 μL of distilled water and homogenized. The resultant supernatant was added to APP-RPA reactions. Of the 15 lung samples positive by qPCR, nine were detected with APP-RPA, a sensitivity of 53.3%. However, the seven samples that failed to amplify with APP-RPA all belonged to the medium and low bacterial burdens, with all eight of the high bacterial load samples amplifying in APP-RPA. Eight clinical samples were paired and could be compared to one another as they were run in all amplification chemistries; qPCR and APP-RPA using extracted DNA, FTA-APP and APP-RPA with lung homogenate, and the results are summarized in Table 3.

**Table 3:** Performance of different extraction methods in APP-RPA.

**Bacterial burden**	**qPCR copies**	**APP-RPA TTP (mins)**		
		**Extracted**	**FTA**	**Homogenate**
High	82,770	2.63	Negative	2.86
	24,300	3.02	1.54	3.73
	21,284	2.78	6.81	3.39
	11,169	3.51	4.33	4.51
Medium	572	5.22	Negative	Negative
	246	6.15	2.97	Negative
Low	185	6.27	5.69	Negative
Negative	0	Negative	Negative	Negative

*Eight clinical lung samples were extracted for gDNA, homogenized and smears made on FTA cards. The FTA cards were processed by 2 5-min washes in deionized water. The 3 mm FTA card discs were subsequently added directly into APP-RPA reactions for detection. Time to positive (TTP) in min of APP-RPA detection compared across the three (extracted gDNA, lung homogenate and FTA card) sample preparation methods*.

## Discussion

In this study, we have described a rapid, simple, sensitive, and specific diagnostic assay targeting the species-specific *apxIVA* gene. Respiratory infections continue to be one of the most common infectious disease burdens within the swine industry (28) and can be caused by a variety of bacterial and/or viral pathogens, collectively known as the porcine respiratory disease complex (29). Knowing which pathogen(s) are involved informs appropriate treatment. Acute porcine pleuropneumonia, caused by APP, can present suddenly in susceptible pigs and, as it is highly contagious, and can result in high morbidity and mortality. Timely diagnosis not only limits the spread of disease but also antibiotic usage and economic losses. Currently, available APP diagnostic methods are labor-intensive, time-consuming, and expensive. Therefore, our relatively inexpensive and rapid APP-RPA assay has a huge potential in the application of point-of-care diagnostics for this important respiratory pathogen.

A bottleneck for RPA assay design is the lack of proprietary software for designing appropriate primers and probes. Therefore, the design of assays must be performed manually to achieve the most efficient primer/probe combination. We initially screened 20 primers (10 forward and 10 reverse) and then selected a single combination which displayed the fastest time to positive result. Several primer properties have been implicated in RPA recombination efficiency, which greatly increases the reaction time. Firstly, longer primers (30–38 bp) promote more efficient recombination, although shorter (e.g., 18 bp) primers (30) and those designed for PCR (31, 32) have been shown to work in RPA. Secondly, as for PCR, 3′ guanine and cytosine anchors are beneficial. Thirdly, GC content should be between 30–70%, and repetitive elements of tandem repeats should be avoided to decrease the likelihood of primer dimerization events.

The dual-labeled RPA exonuclease probes require internal modifications, as opposed to qPCR probes which are typically labeled at 3′ and 5′ ends. Thus, RPA probes are relatively expensive and must be designed with great care. Recently an RPA based assay (33) was described for detection of the APP *apxIVA* gene which utilized the ZC BioScience^TM^ Exo kit. However, we found that the primer-probe combination used in that study was not able to detect all APP serovar reference strains using the TwistDx reaction format. Specifically, serovars 9 and 11 were not detected due to a 16-base deletion in the target region of the designed probe (data not shown).

We verified that our APP-RPA assay detected all 19 currently known serovars of APP, which is (to our knowledge) the first isothermal assay to have achieved this. Furthermore, all serovars amplified rapidly (under 6 min at 200 copies/μL) with no significant difference in time-to-positive. This suggests that our *apxIVA* region is highly conserved between all currently known APP serovars. Whilst our APP-RPA assay allows rapid detection of the presence of APP (via the species-specific *apxIVA* gene), determination of the relevant serovar (important for epidemiological surveillance and for informing vaccination strategies) would require further testing of positive samples, currently provided at centralized diagnostic laboratories.

Although our APP-RPA gave positive amplification for all 19 serovars, the *apxIVA* gene is not 100% conserved between isolates, and insertions such as IS*Apl1* have been detected in the *apxIVA* gene in some isolates, which could affect detection (34). When tested with isolates that have been found to have a transposase adjacent to or within their *apxIVA* genes, we found the isolate MIDG3936 with a WP_005599960 transposon flanking the *apxIVA* gene duplication (*apxIVS'*) and a fragmented *apxIVA* gene, had a similar detection time to the reference serovar 2 strain. This indicates that the fragmentation of the *apxIVA* gene did not interfere with detection by APP-RPA, or that *apxIVS'* rescued any sensitivity that may be caused by the fragmentation of the *apxIVA* gene. The isolate MIDG2206, containing a hypothetical protein (WP-017357847) between the *apxIVA* and *lacZ* genes, also resulted in near-identical amplification as the reference serovar 15 strain. Therefore, this untypical arrangement of the genes immediately adjacent to the *apxIVA* gene does not result in any loss in sensitivity in the APP-RPA assay and is unlikely to result in any false-negative results in clinical samples. However, the forward and reverse primers in the qPCR assay used in this study target a different region of the *apxIVA* gene (GenBank: LN908249.1 nt: 1,124,391-1,124,767). Therefore, it would be difficult to ascertain the true clinical sensitivity of our APP-RPA assay as an insertion in the *apxIVA* gene in the target region may prevent detection in the qPCR assays. Thus, a relative clinical sensitivity of our APP-RPA assay was determined against a previously validated *apxIVA* qPCR assay (16), using a collection of clinical samples originating from England and Portugal. Of the 61 clinical samples from pigs with suspected pleuropneumonia, 36 samples originated from farms in England, where serovar 8 is known to predominate (9). Of the 36 English samples, three were APP negative, 16 had low, nine had medium, and eight had high APP burden, as determined by qPCR. Comparative results with our APP-RPA assay indicated an overall clinical sensitivity of 76.5% in these English isolates. The other 25 clinical samples were obtained from Portuguese farms, where serovars 5 and 17 predominate (unpublished data). Of these samples (all lung tissue), seven were negative, two had low, six had medium, and 10 had high APP burden, as determined by qPCR. Comparatively, our APP-RPA achieved a sensitivity of 100% with these Portuguese samples.

Overall, our APP-RPA assay achieved a sensitivity of 84.3% for the 61 clinical samples. However, when relative quantification of copy number, as determined by qPCR, was considered, the false-negative samples in APP-RPA were found to be largely due to samples with low copy number (<200 copies), specifically samples with <55 gDNA copies per μL. Only 10 of the 18 low copy lung samples gave positive amplification with APP-RPA. The high proportion of lung samples harboring low APP burden may be indicative of sampling artifacts seen when sampling tissue with a non-uniform distribution of bacteria. Alternatively, a low APP burden may indicate presence of gDNA as a result of a previous APP infection. However, little is known about the rate at which the presence of APP gDNA wanes during the course of APP infections.

Current evidence suggests that colonization of the tonsils plays a role in disease transmission (35), as APP is known to be harbored in tonsillar crypts of sub-clinically-infected pigs, including those which have survived acute lung infection (36, 37). Asymptomatic carriage of APP is thought to be responsible for the introduction of infections into naive herds, and for sporadic recurrent disease presentation. However, detection of this bacterium in oral fluid samples is known to be problematic (38), with some evidence of serovar differences in the ability to colonize the oral cavity (39). Furthermore, antimicrobial treatment has been shown not to significantly clear carriage in the tonsils (40). Therefore, there is a need to have a diagnostic tool that can detect sub-clinically, as well as clinically infected swine (8). To ascertain the feasibility of detecting sub-clinically infected animals, we obtained five oral fluid samples from UK farms. All five of the oral fluid samples were positive for APP, with one high, and four low bacterial loads of APP - with qPCR quantification at 260, 133, 115, 111, and 82 copies/μ*L*. All five oral samples were detected in APP-RPA in under 7 min. The rapid time to positive and relatively late Ct obtained in qPCR of the oral fluid samples is unusual, as each sample represented a pool of 25 animals. This inconsistency in results may be due to inhibition or contaminants in the oral sample (such as other bacterial species), as RPA has been shown to be less prone to common PCR inhibitors (41, 42). Given the higher sensitivity of qPCR, it is encouraging that we were able to detect all five of the positive oral fluid samples with our APP-RPA, which is faster and more amenable to point of care use. However, the lack of overall sensitivity of our APP-RPA at low bacterial loads suggests, this technique is not suitable to detect asymptomatic carrier animals. Furthermore, the ability of our APP-RPA to sensitively detect medium and high copy numbers of APP suggests it is more suitable for deployment as a rapid point-of-care assay, especially where APP is suspected from the clinical presentation and where rapid diagnosis would have the potential to limit spread through swift treatment and isolation of contact animals.

Other isothermal techniques, such as loop-mediated isothermal amplification (LAMP) assays (43, 44), have been developed for the detection of APP. However, LAMP requires a higher temperature to operate than RPA (60–65°C rather than 37°C), has a more complex primer design, and is not currently commercially available in a lyophilized format, which is essential for use in the field in a point-of-care scenario. Therefore, LAMP is more difficult to incorporate into low-resource field settings. RPA has already been taken into remote settings, with some groups describing a simple “lab in a suitcase” for detection of pathogens responsible for human epidemics, including Ebola and SARS CoV-2 (45, 46).

A major challenge in point-of-care diagnostics is the extraction of samples in resource limited settings, with more commercial extraction kits requiring high powered table-top centrifuges, which are not mobile. The possibility to directly amplify from FTA cards with RPA would significantly simplify this extraction bottleneck. Our previously described FTA-multiplex-PCR for APP detection was based on imprinting the FTA card on lung tissue (visibly lesioned areas, where possible) and two 5-min water washes (23). As lung samples have been previously optimized for use of FTA cards and are the most commonly collected sample type collected for APP diagnostic investigations, we did not seek to validate other tissue types on FTA cards.

Our current small-scale validation using FTA cards in combination with the APP-RPA assay showed a sensitivity and specificity of 76.9 and 100%, respectively. When compared to results using extracted gDNA from paired lung samples (*n* = 10), all 10 were detected in extracted samples, however two of these samples failed to be detected in FTA-APP-RPA. Only one of the three lung samples quantified as having low bacterial load in extracted lung tissue failed to be detected in FTA-APP-RPA. Surprisingly, one sample containing high and one containing medium bacterial load (82,770 and 572 copies, respectively) also failed to be detected. This may be due to inconsistencies in sampling, and repeated sampling of different areas of the lung may increase the probability of sampling where the bacteria are present. This requires further investigation but is beyond the scope of the current study. However, the decrease in sensitivity when FTA cards were used in combination with RPA suggests that sub-clinically infected animals with low bacterial loads will be missed with this rapid method of detection. The use of a rapid processing method for lung tissue would facilitate the use of point-of-care techniques on farm, we thus sought to compare the use of gDNA extracted and quantified by qPCR to a crude homogenization of lung tissue would release enough bacterial gDNA to be detected by APP-RPA. The use of homogenized tissues provided a higher correlation than FTA-APP-RPA with qPCR copy numbers, with only the lung samples displaying the lowest copy numbers tested (below 12,000 copies), testing negative with RPA. The achieved APP-RPA detection limit increased from the 55 copies in extracted clinical samples to 12,000 copies in homogenized tissue. Therefore, the use of lung homogenate prioritizes the rapid preparation of samples over assay sensitivity. Overall, the use of FTA-cards or homogenized tissues in conjunction with APP-RPA are feasible, bypassing the labor-intensive and impractical commercial extraction of clinical lung samples.

Several limitations to our current study exist, firstly although *apxIVA* is widely used as an APP species marker, the instance of insertional elements within or distally to the *apxIVA* gene or partial duplicons in this locus makes assay design troublesome. We have used isolates with both transposons and small tandem duplications - which have yet to interfere with our APP-RPA diagnostic. However, it should be noted that duplications of the *apxIVA* gene result in alternative priming sites which can contain single-polynucleotide polymorphisms. Whilst our sequence analysis suggests that at least one perfect match APP-RPA binding region is present within these isolates, the assessment of how alternative priming sites may affect either the qPCR or APP-RPA assays was beyond the scope of this study. Secondly, we determined that our lower limit of detection in clinical samples equates to 55 copies/μL. In order to assess our clinical performance, we grouped our data into negative, low, medium and high bacterial loads based on qPCR relative quantification. We placed our low bacterial load group at less than 200 copies/μL for two reasons; it gave a uniform sample number in each group and the distribution of the qPCR copy numbers of samples gave a natural break across these groups. If we had chosen to classify <55 copies as ultra-low, we would have achieved 100% sensitivity and specificity across all other groups. However, this may lead to the false interpretation of our results as Eight of the 18 low copy number samples were below 55 copies, which may still retain clinical relevance to the APP status of an animal. Thirdly, due to limited availability, our number of oral fluid samples was low (five out of the 61 clinical samples tested). Tonsil samples could have been used; however, no tonsil samples were available for analysis although we envisage that APP-RPA could be applied to such samples. Our limited data suggests that although oral samples contain a relatively low bacterial burden compared to the majority of lung samples, they may still prove a useful tool in detecting sub-clinically infected animals, although more samples are required to provide a full validation. Lastly, we describe the detection of APP in APP-RPA using alternative sampling methods, whilst our FTA cards gave inconsistent results in relation to onset time and the achieved limit of detection. We used qPCR of extracted tissue from the same lung tissue as our “gold standard” to determine the copy number. However, the presence of bacteria may not be uniform across all the tissue areas, and the use of different sampling areas for each method may have accounted for the discrepancies seen in the ability to detect APP with APP-RPA.

In conclusion, we have developed an APP-RPA assay that is rapid and specific for detecting all 19 known serovars of APP. Furthermore, the APP-RPA displays good sensitivity using either extracted gDNA, homogenized samples, or imprinted on FTA-cards. Our data suggests that an RPA-based field deployable diagnostic test for APP enabling the rapid detection and screening of this highly economically important pathogen is feasible.

## Data Availability Statement

The raw data supporting the conclusions of this article will be made available by the authors, without undue reservation.

## Author Contributions

OS and MF designed and optimized RPA assay conditions. OS and YL designed and performed experiments including data analysis. OS, JB, YL, and PL all contributed to writing the manuscript. JH-G, AT, FC, PM, EV, and PP provided clinical samples. MF, JH-G, AT, FC, PM, EV, PP, and JR-M were all involved in proof-reading the manuscript. PL and PG secured funding. All authors: study concept. All authors contributed to the article and approved the submitted version.

## Funding

Grants from the Biotechnology and Biological Sciences Research Council (BB/S002103/1 and BB/S005897/1). OS was supported by a BBSRC-funded DTP studentship (BB/M011178/1).

## Conflict of Interest

TN, PM, EV, and PP are employed by Ceva Animal Health Ltd. The remaining authors declare that the research was conducted in the absence of any commercial or financial relationships that could be construed as a potential conflict of interest.

## Publisher's Note

All claims expressed in this article are solely those of the authors and do not necessarily represent those of their affiliated organizations, or those of the publisher, the editors and the reviewers. Any product that may be evaluated in this article, or claim that may be made by its manufacturer, is not guaranteed or endorsed by the publisher.

## References

[1] Gottschalk M Broes A. “Actinobacillosis,” in Disease of Swine. 11th ed. In: Zimmerman JJ, Karriker LA, Ramirez A, Schwartz KJ, Stevenson GW, Zhang J, editors. Hoboken, NJ: John Wiley & Sons, Inc., (2019). p. 749–766.

[2] Gale C Velazquez E. *Actinobacillus pleuropneumoniae*: a review of an economically important pathogen. Livestock. (2020) 25:6. 10.12968/live.2020.25.6.3082657061

[3] Bossé JT Janson H Sheehan BJ Beddek AJ Rycroft AN Kroll JS. *Actinobacillus pleuropneumoniae*: pathobiology and pathogenesis of infection. Microbes Infect. (2002) 4:225–35. 10.1016/S1286-4579(01)01534-9 11880056

[4] Bossé JT Li Y Rogers J Fernandez Crespo R Li Y Chaudhuri RR. Whole genome sequencing for surveillance of antimicrobial resistance in *Actinobacillus pleuropneumoniae*. Front Microbiol. (2017) 8:6–11. 10.3389/fmicb.2017.00311 28321207 PMC5337627

[5] Kim B Hur J Lee JY Choi Y Lee JH. Molecular serotyping and antimicrobial resistance profiles of *Actinobacillus pleuropneumoniae* isolated from pigs in South Korea. Vet Q. (2016) 36:137–44. 10.1080/01652176.2016.1155241 26879953

[6] Holmer I Salomonsen CM Jorsal SE Astrup LB Jensen VF Høg BB. Antibiotic resistance in porcine pathogenic bacteria and relation to antibiotic usage. BMC Vet Res. (2019) 15:1–13. 10.1186/s12917-019-2162-8 31829171 PMC6907208

[7] Stringer OW Bossé JT Lacouture S Gottschalk M Fodor L Angen Ø. Proposal of *Actinobacillus pleuropneumoniae* serovar 19, and reformulation of previous multiplex PCRs for capsule-specific typing of all known serovars. Vet Microbiol. (2021) 255:109021. 10.1016/j.vetmic.2021.109021 33667982

[8] Sassu EL Bossé JT Tobias TJ Gottschalk M Langford PR Hennig-Pauka I. Update on *Actinobacillus pleuropneumoniae*- knowledge, gaps and challenges. Transbound Emerg Dis. (2018) 65:72–90. 10.1111/tbed.12739 29083117

[9] Li Y Bossé JT Williamson SM Maskell DJ Tucker AW Wren BW. *Actinobacillus pleuropneumoniae* serovar 8 predominates in England and Wales. Vet Rec. (2016) 179:8–9. 10.1136/vr.103820 27531715 PMC5036230

[10] Mittal KR Bourdon S. Cross-reactivity and antigenic heterogeneity among *Actinobacillus pleuropneumoniae* strains of serotypes 4 and 7. J Clin Microbiol. (1991) 29:1344–7. 10.1128/jcm.29.7.1344-1347.1991 1909343 PMC270113

[11] Mittal KR. Cross-reactions between *Actinobacillus (Haemophilus) pleuropneumoniae* strains of serotypes 1 and 9. J Clin Microbiol. (1990) 28:535–9. 10.1128/jcm.28.3.535-539.1990 1691210 PMC269658

[12] Gottschalk M. The challenge of detecting herds sub-clinically infected with *Actinobacillus pleuropneumoniae*. Vet J. (2015) 206:30–8. 10.1016/j.tvjl.2015.06.016 26206322

[13] Bossé JT Li Y Fernandez Crespo R Lacouture S Gottschalk M Sárközi R Fodor L. Comparative sequence analysis of the capsular polysaccharide loci of *Actinobacillus pleuropneumoniae* serovars 1–18, and development of two multiplex PCRs for comprehensive capsule typing. Vet Microbiol. (2018) 220:83–9. 10.1016/j.vetmic.2018.05.011 29885806 PMC6008488

[14] Schaller A Djordjevic SP Eamens GJ Forbes WA Kuhn R Kuhnert P. Identification and detection of *Actinobacillus pleuropneumoniae* by PCR based on the gene *apxIVA*. Vet Microbiol. (2001) 79:47–62. 10.1016/S0378-1135(00)00345-X 11230928

[15] Sthitmatee N Sirinarumitr T Makonkewkeyoon L Sakpuaram T Tesaprateep T. Identification of the *Actinobacillus pleuropneumoniae* serotype using PCR based-*apx* genes. Mol Cell Probes. (2003) 17:301–5. 10.1016/j.mcp.2003.08.001 14602481

[16] Tobias TJ Bouma A Klinkenberg D Daemen AJJM Stegeman JA Wagenaar JA. Detection of *Actinobacillus pleuropneumoniae* in pigs by real-time quantitative PCR for the *apxIVA* gene. Vet J. (2012) 193:557–60. 10.1016/j.tvjl.2012.02.004 22445313

[17] Piepenburg O Williams CH Stemple DL Armes NA. DNA. detection using recombination proteins. PLoS Biol. (2006) 4:1115–21. 10.1371/journal.pbio.0040204 16756388 PMC1475771

[18] Formosa T Alberts BM. Purification and characterization of the T4 bacteriophage uvsX protein. J Biol Chem. (1986) 261:6107–18. 10.1016/S0021-9258(17)38499-5 2939071

[19] Liu L Li R Zhang R Wang J An Q Han Q. Rapid and sensitive detection of *Mycoplasma hyopneumoniae* by recombinase polymerase amplification assay. J Microbiol Methods. (2019) 159:56–61. 10.1016/j.mimet.2019.02.015 30807776

[20] Yang Y Qin X Sun Y Chen T Zhang Z. Rapid detection of highly pathogenic porcine reproductive and respiratory syndrome virus by a fluorescent probe-based isothermal recombinase polymerase amplification assay. Virus Genes. (2016) 52:883–6. 10.1007/s11262-016-1378-y 27534870

[21] Zhang T Liu M Yin R Yao L Liu B. Rapid and simple detection of *Glaesserella parasuis* in synovial fluid by recombinase polymerase amplification and lateral flow strip. BMC Vet Res. (2019) 294:1–7. 10.1186/s12917-019-2039-x 31412870 PMC6694577

[22] Zhao G He H Wang H. Use of a recombinase polymerase amplification commercial kit for rapid visual detection of *Pasteurella multocida*. BMC Vet Res. (2019) 15:1–8. 10.1186/s12917-019-1889-6 31101109 PMC6525368

[23] Stringer OW Bossé JT Lacouture S Gottschalk M Fodor L Angen Ø. Rapid detection and typing of *Actinobacillus pleuropneumoniae* serovars directly from clinical samples : combining FTA^®^ card technology with multiplex PCR. Front Vet Sci. (2021) 8:1–9. 10.3389/fvets.2021.728660 34447805 PMC8382971

[24] Srijuntongsiri G Mhoowai A Samngamnim S Assavacheep P Bossé JT Langford PR. Novel DNA markers for identification of *Actinobacillus pleuropneumoniae*. Microbiol Spectr. (2022) 10:e0131121. 10.1128/spectrum.01311-21 34985298 PMC8729771

[25] Nielsen R. O'connor PJ. Serological characterization of 8 *Haemophilus pleuropneumoniae* strains and proposal of a new serotype: serotype 8. Acta Vet Scand. (1984) 25:96–106. 10.1186/BF03547283 6464928 PMC8287466

[26] Lillis L Siverson J Lee A Cantera J Parker M Piepenburg O. Factors influencing recombinase polymerase amplification (RPA) assay outcomes at point of care. Mol Cell Probes. (2016) 30:1247–62. 10.1016/j.mcp.2016.01.009 26854117 PMC4818709

[27] Hernandez-Garcia J Robben N Magnée D Eley T Dennis I Kayes SM. The use of oral fluids to monitor key pathogens in porcine respiratory disease complex. Porc Health Manag. (2017) 3:1–13. 10.1186/s40813-017-0055-4 28405463 PMC5382517

[28] USDA. Swine 2012: Part II: Reference of Swine Health and Health Management in the United States, 2012. USDA (2016).

[29] Opriessnig T Giménez-Lirola LG Halbur PG. Polymicrobial respiratory disease in pigs. Anim Health Res Rev. (2011) 12:133–48. 10.1017/S1466252311000120 22152290

[30] Fuller SL Savory EA Weisberg AJ Buser JZ Gordon MI Putnam ML. Isothermal amplification and lateral-flow assay for detecting crown-gall-causing *Agrobacterium* spp. Phytopathology. (2017) 107:1062–8. 10.1094/PHYTO-04-17-0144-R 28569126

[31] Mayboroda O Benito AG Del Rio JS Svobodova M Julich S Tomaso H. Isothermal solid-phase amplification system for detection of *Yersinia pestis*. Anal Bioanal Chem. (2016) 408:671–6. 10.1007/s00216-015-9177-1 26563112

[32] Wang R Zhang F Wang L Qian W Qian C Wu J. Instant, visual, and instrument-free method for on-site screening of GTS 40-3-2 soybean based on body-heat triggered recombinase polymerase amplification. Anal Chem. (2017) 89:4413–8. 10.1021/acs.analchem.7b00964 28345860

[33] Li R Wang J Liu L Zhang R Hao X Han Q. Direct detection of *Actinobacillus pleuropneumoniae* in swine lungs and tonsils by real-time recombinase polymerase amplification assay. Mol Cell Probes. (2019) 45:14–8. 10.1016/j.mcp.2019.03.007 30930280

[34] O'Neill C Jones SCP Bossé JT Watson CM Williamson SM Rycroft AN. Population-based analysis of *Actinobacillus pleuropneumoniae* ApxIVA for use as a DIVA antigen. Vaccine. (2010) 28:4871–4. 10.1016/j.vaccine.2010.04.113 20483193 PMC4843962

[35] Velthuis AGJ De Jong MCM Kamp EM Stockhofe N Verheijden JHM. Design and analysis of an *Actinobacillus pleuropneumoniae* transmission experiment. Prev Vet Med. (2003) 60:53–68. 10.1016/S0167-5877(03)00082-5 12900149

[36] Hoeltig D Nietfeld F Strutzberg-Minder K Rohde J. Evaluation of the predictive value of tonsil examination by bacteriological culture for detecting positive lung colonization status of nursery pigs exposed to *Actinobacillus pleuropneumoniae* by experimental aerosol infection. BMC Vet Res. (2018) 14:10–6. 10.1186/s12917-018-1542-9 29954395 PMC6022346

[37] Sassu EL Ladinig A Talker SC Stadler M Knecht C Stein H. Frequency of Th17 cells correlates with the presence of lung lesions in pigs chronically infected with *Actinobacillus pleuropneumoniae*. Vet Res. (2017) 48:1–16. 10.1186/s13567-017-0411-z 28166835 PMC5294905

[38] Goecke NB Kobberø M Kusk TK Hjulsager CK Pedersen KS Kristensen CS. Objective pathogen monitoring in nursery and finisher pigs by monthly laboratory diagnostic testing. Porc Health Manag. (2020) 6:1–14. 10.1186/s40813-020-00161-3 32922832 PMC7476771

[39] Costa G. Oliveira S, Torrison J. Detection of *Actinobacillus pleuropneumoniae* in oral-fluid samples obtained from experimentally infected pigs. J Swine Health Prod. (2012) 20:78–81. 10.1515/jvetres-2017-0021 29978069 PMC5894388

[40] Angen Ø Andreasen M Nielsen EO Stockmarr A Bækbo P. Effect of tulathromycin on the carrier status of *Actinobacillus pleuropneumoniae* serotype 2 in the tonsils of pigs. Vet Rec. (2008) 163:445–7. 10.1136/vr.163.15.445 18849576

[41] Rosser A Rollinson D Forrest M Webster BL. Isothermal recombinase polymerase amplification (RPA) of *Schistosoma haematobium* DNA and oligochromatographic lateral flow detection. Parasit Vectors. (2015) 8:1–5. 10.1186/s13071-015-1055-3 26338510 PMC4559068

[42] Eid C Santiago JG. Assay for: *Listeria monocytogenes* cells in whole blood using isotachophoresis and recombinase polymerase amplification. Analyst. (2017) 142:48–54. 10.1039/C6AN02119K 27904893

[43] Yang W Pin C Haibing G Yang C Hui L Qigai H. Loop-mediated isothermal amplification targeting the *apxIVA* gene for detection of *Actinobacillus pleuropneumoniae*. FEMS Microbiol Lett. (2009) 300:83–9. 10.1111/j.1574-6968.2009.01779.x 19765085

[44] Ji H Haitao-Li Zhu L Zhang H Wang Y Zuo Z. Development and evaluation of a loop-mediated isothermal amplification (LAMP) assay for rapid detection of *Actinobacillus pleuropneumoniae* based the dsbE-like gene. Pesqui Vet Bras. (2012) 32:757–60. 10.1590/S0100-736X2012000800014

[45] El Wahed AA Patel P Maier M Pietsch C Rüster D Böhlken-Fascher S. Suitcase lab for rapid detection of SARS-CoV-2 based on recombinase polymerase amplification assay. Anal Chem. (2021) 93:2627–34. 10.1021/acs.analchem.0c04779 33471510 PMC7839158

[46] Faye O Faye O Soropogui B Patel P El Wahed AA Loucoubar C. Development and deployment of a rapid recombinase polymerase amplification Ebola virus detection assay in Guinea in 2015. Eurosurveillance. (2015) 20:2. 10.2807/1560-7917.ES.2015.20.44.30053 26558690

