# Supplementary material for: Rapid Detection of Actinobacillus pleuropneumoniae From Clinical Samples Using Recombinase Polymerase Amplification
Source: Front Vet Sci. 2022 Mar 25;9:805382. doi: 10.3389/fvets.2022.805382 (PMC8990124; doi:10.3389/fvets.2022.805382)
Supplement: Supplementary file 1 [file Presentation_1.PPTX]

## Slide 1
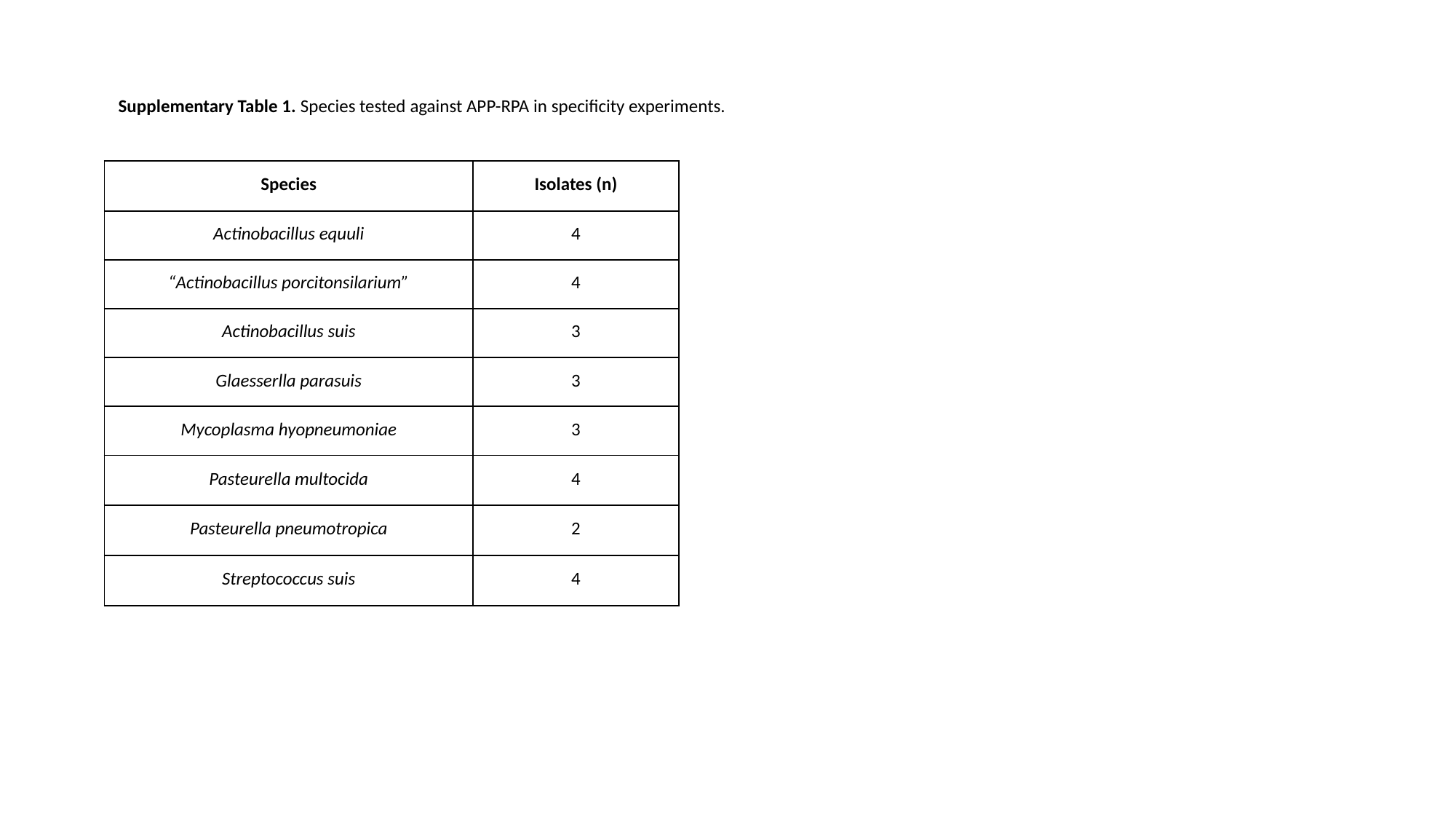

Supplementary Table 1. Species tested against APP-RPA in specificity experiments.
| Species | Isolates (n) |
| --- | --- |
| Actinobacillus equuli | 4 |
| “Actinobacillus porcitonsilarium” | 4 |
| Actinobacillus suis | 3 |
| Glaesserlla parasuis | 3 |
| Mycoplasma hyopneumoniae | 3 |
| Pasteurella multocida | 4 |
| Pasteurella pneumotropica | 2 |
| Streptococcus suis | 4 |

## Slide 2
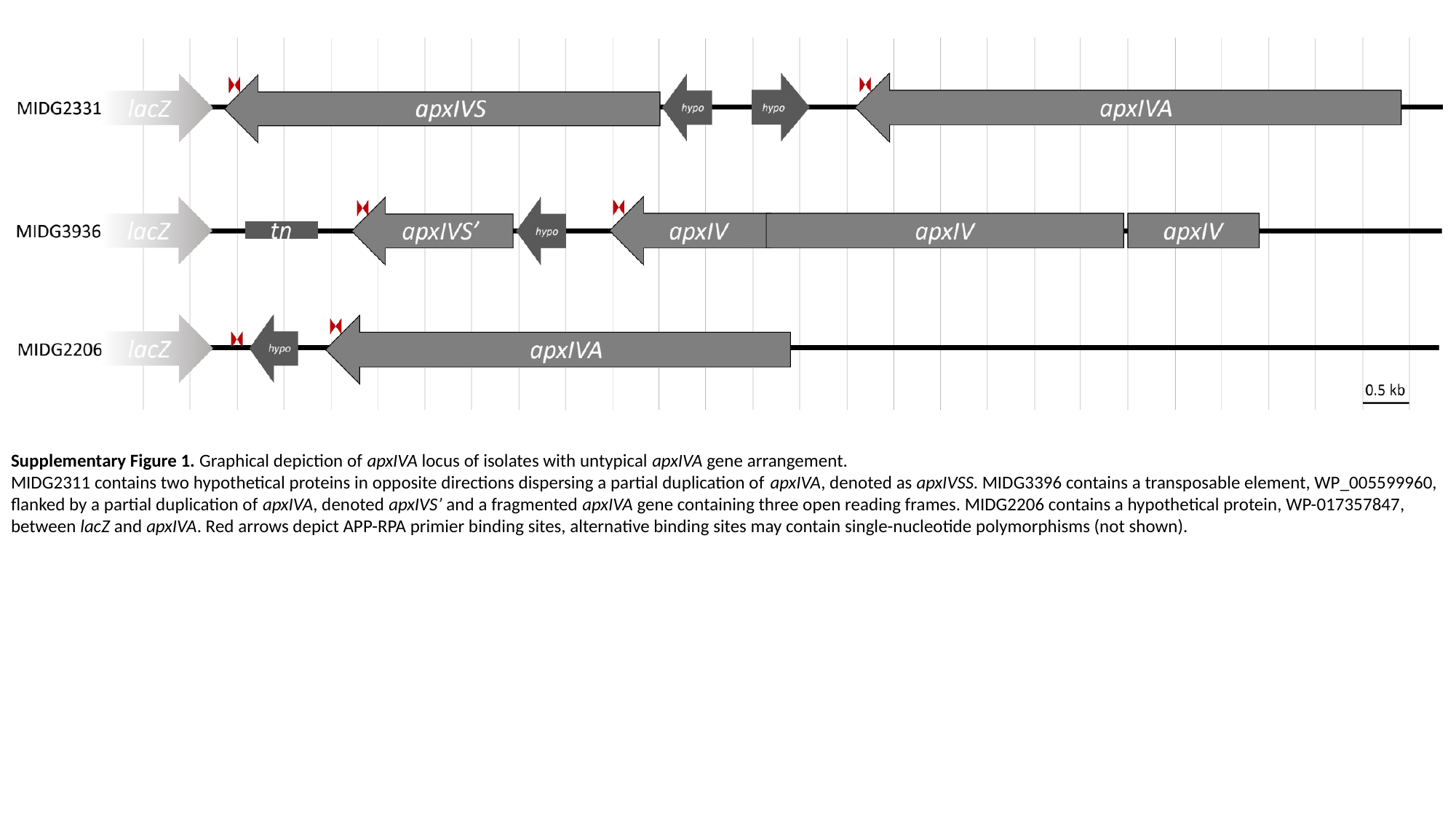

Supplementary Figure 1. Graphical depiction of apxIVA locus of isolates with untypical apxIVA gene arrangement.
MIDG2311 contains two hypothetical proteins in opposite directions dispersing a partial duplication of apxIVA, denoted as apxIVSS. MIDG3396 contains a transposable element, WP_005599960, flanked by a partial duplication of apxIVA, denoted apxIVS’ and a fragmented apxIVA gene containing three open reading frames. MIDG2206 contains a hypothetical protein, WP-017357847, between lacZ and apxIVA. Red arrows depict APP-RPA primier binding sites, alternative binding sites may contain single-nucleotide polymorphisms (not shown).
